# Maternal Serum and Breast Milk Vitamin D Levels: Findings from the Universiti Sains Malaysia Pregnancy Cohort Study

**DOI:** 10.1371/journal.pone.0100705

**Published:** 2014-07-03

**Authors:** Hamid Jan Jan Mohamed, Angela Rowan, Bertram Fong, See-Ling Loy

**Affiliations:** 1 Nutrition Program, School of Health Sciences, Universiti Sains Malaysia, Kubang Kerian, Kelantan, Malaysia; 2 Fonterra Co-operative Group Ltd, Palmerston North, New Zealand; 3 Fonterra Research and Development Centre, Palmerston North, New Zealand; Chiba University Center for Forensic Mental Health, Japan

## Abstract

**Background:**

Vitamin D deficiency has become a global health issue in pregnant women. This study aimed to assess the adequacy of maternal vitamin D status by measuring maternal serum and breast milk 25-hydroxyvitamin D [25(OH)D] levels and to determine the association between maternal serum and milk 25(OH)D levels.

**Methods:**

Data was obtained from the Universiti Sains Malaysia Pregnancy Cohort Study. This study was conducted from April 2010 to December 2012 in the state of Kelantan, Malaysia. Blood samples from pregnant women aged 19 to 40 years were drawn in the second and third trimesters of pregnancy, while breast milk samples at delivery, 2, 6 and 12 months postpartum were collected to analyze for 25(OH)D levels. A total of 102 pregnant women were included in the analysis.

**Results:**

Vitamin D deficiency [25(OH)D <50 nmol/L] was detected in 60% and 37% of women in the second and third trimesters of pregnancy, respectively. There were 6% and 23% of women who reached normal level of vitamin D status in the second trimester and the third trimester, respectively. Multivitamin intakes during pregnancy were significantly associated with higher serum 25(OH)D levels in the second trimester (β = 9.16, p = 0.005) and the third trimester (β = 13.65, p = 0.003). 25(OH)D levels in breast milk during the first year of lactation ranged from 1.01 to 1.26 nmol/L. Higher maternal serum 25(OH)D level in the second trimester of pregnancy was associated with an elevated level of 25(OH)D in breast milk at delivery (β = 0.002, p = 0.026).

**Conclusions:**

This study shows that high proportions of Malay pregnant women are at risk of vitamin D deficiency. Maternal vitamin D status in the second trimester of pregnancy was found to influence vitamin D level in breast milk at delivery.

## Introduction

The role of vitamin D in health outcomes related to pregnancy, the perinatal period and young children has received considerable interest recently. Although evidence is inconsistent, numerous studies have reported that low maternal vitamin D status is associated with multiple adverse obstetric outcomes and thereby, is increasingly recognized as a global health problem [1]. Vitamin D deficiency during pregnancy has been linked with maternal osteomalacia, gestational diabetes, preeclampsia, small birth size, respiratory diseases, impaired fetal growth and bone development later in childhood [1]–[4], and more recently adequate vitamin D status has been linked to fetal neurodevelopment [5], [6].

The major circulating form of vitamin D in blood is 25-hydroxyvitamin D [25(OH)D]. Serum 25(OH)D is currently accepted as the best biochemical indicator of vitamin D status [7]. However, the level of circulating 25(OH)D required for optimal health is uncertain, and the normal range of 25(OH)D concentration in pregnancy and lactation is unknown [3], [8]. Few guidelines have been established in defining the cut-off point for vitamin D status [9]–[12]. Overall, most researchers agree that serum 25(OH)D levels below 50 nmol/L are defined as deficiency [13]–[15]. It has been reported that both maternal and infant complications associated with low vitamin D occur more often with a serum vitamin D level below 50 nmol/L [2]. The cut-off that defines vitamin D insufficiency is based on a threshold for serum 25(OH)D above which there is no further suppression of parathyroid hormone (PTH) to reduce bone loss [16], [17]. It is suggested that poor calcium intakes require higher 25(OH)D levels to exert maximal suppression of PTH [17].

Breast milk is considered the optimal source of nutrition during early infancy. There are, however, increasing reports that exclusively breastfed infants with inadequate sunlight exposure and without vitamin D supplementation have an increased risk of rickets [18], [19]. Although human milk is generally thought to be a poor source of vitamin D [7], there is a need to reassess breast milk vitamin D levels and factors that may affect this in order to investigate a strategy to optimize levels of vitamin D in breast milk and thus improve the vitamin D status of the breastfed infant [19]. This study therefore aimed (i) to examine maternal serum and breast milk 25(OH)D levels; and (ii) to associate 25(OH)D levels in breast milk during the first year postpartum with maternal values during pregnancy.

## Materials and Methods

### Ethics statement

The present data was obtained from the Universiti Sains Malaysia (USM) Pregnancy Cohort Study which was conducted between April 2010 and December 2012 in Kelantan, Malaysia [20]. The study protocol was approved by the Human Research Ethics Committee of USM and Medical Research Ethics Committee of Ministry of Health, Malaysia.

### Study design and participants

A subsample of pregnant women (n = 102) from the cohort with complete data on 25(OH)D analysis were used in this study. Pregnant women were recruited from the Obstetrics and Gynecology (O & G) Clinic of Hospital Universiti Sains Malaysia (HUSM) and Kubang Kerian Health Clinic, Kota Bharu. Convenience sampling technique was adopted for sample selection. The inclusion criteria were defined as i) Malaysian and Malay ethnicity, ii) aged 19 to 40 years, iii) singleton pregnancy, iv) gestational age 24 weeks and less based on the last menstrual period or early ultrasound examination, v) plan to give birth in HUSM, and vi) live within a distance of 50 km from HUSM. Exclusion criteria included i) diagnosed with pre-existing chronic diseases or pregnancy complications and ii) preterm delivery before 37 weeks of gestation. The study objective and procedure were explained to the eligible women and the written informed consent documents were signed. These recruited women were followed from the second trimester (14–24 gestational weeks) to the third trimester of pregnancy (≥32 gestational weeks), 1 to 14 days after giving birth, 2, 6 and 12 months postpartum. Data on socio-demographic characteristics, obstetrics history, smoking status, physical activity, dietary intake, multivitamin intake that included vitamin B complex, vitamin C, folic acid, iron and vitamin D supplements during pregnancy were collected using questionnaires via interview.

### Anthropometry

Maternal height was measured with a microtoise tape (Seca 206, Hamburg, Germany) while pre-pregnancy weight was based on the maternal recall. Body mass index (BMI) was computed from maternal weight (kg)/height (m^2^).

### Dietary assessment

Maternal dietary calcium intake was assessed using 24-hour diet recalls in the second and third trimesters of pregnancy. A four stage, multiple-pass interviewing technique was used to conduct the diet recalls [21]. Atlas of Food Exchanged & Portion Sizes was used to aid in quantifying food portion sizes [22]. Dietary calcium intake was derived using the Nutritionist Pro Diet Analysis software (Axxya Systems LLC., USA) based on Nutrient Composition of Malaysian Foods database and U.S. Department of Agriculture (USDA) Foods database.

### Physical activity

A short form of the International Physical Activity Questionnaire (IPAQ) was used to assess maternal physical activity in the second and third trimesters of pregnancy. Three specific types of activity were included in the questionnaire, which were walking, moderate-intensity activities and vigorous-intensity activities. Total activity score was computed from the summation of the duration (in minutes) and frequency (days) of these three types of activity. The score was expressed in metabolic equivalents (Mets-minutes/week) and categorized as low, moderate and high levels of physical activity [23].

### 25(OH)D analysis

A fasting venous blood sample was collected from each woman in the second and third trimesters of pregnancy. These blood samples were centrifuged at 3500 rpm for 10 min at 4°C (Eppendorf Centrifuge 5810R, Hamburg, Germany). Breast milk samples were collected using a mini electric breast pump (Medela, Illinous, USA) within 1–14 days after delivery, at 2, 6 and 12 months postpartum. Both serum and breast milk samples were stored in −80°C freezer (Thermo Fisher Scientific 702, USA) until analysis. The 25(OH)D analysis was performed by High Performance Liquid Chromatography (HPLC) with a C8 column coupled to an API 3200 Q-trap mass spectrometer (MS, AB SCIEX Framingham, MA, USA), as described by Lewis and Elder [24]. The limit of quantification was at ∼2 nmol/L. The serum and breast milk 25(OH)D results were expressed in nmol/L. Breast milk 25(OH)D levels were also shown in IU/L (40 IU/L = 1 ug/L, MW = 404 g/mol) for the purpose of comparison with other studies [19]. Depending on maternal serum 25(OH)D levels, the women were categorized as severe vitamin D deficiency (<25 nmol/L), mild vitamin D deficiency (25–<50 nmol/L), vitamin D insufficiency (50–<75 nmol/L) and vitamin D sufficiency (≥75 nmol/L).

### Statistical Analysis

Data was analyzed using IBM SPSS statistics, Version 19.0 (USA). Difference in serum 25(OH)D levels between second and third trimesters of pregnancy was compared using paired t-test. Differences in serum 25(OH)D levels according to maternal factors were compared using One-Way ANOVA. Differences in breast milk 25(OH)D levels during the first year lactation were compared using repeated measures ANOVA. Multiple linear regression analysis with confounders forcibly entered was used to examine the association between maternal factor (independent variable) and serum 25(OH)D level (dependent variables), and the association between maternal serum (independent variable) and breast milk 25(OH)D levels (dependent variable). Skewed data were log-transformed to reduce the influence of outliers. Finding at *p*<0.05 for a two-sided test was considered statistically significant.

## Results

Table 1 shows the distributions of pregnant women by characteristics. The mean age of the women was 29.11 years (SD 4.50). Most women were multiparous (42.2%), employed (78.4%) and within normal BMI status (57.8%). Less than 30% of them were supplemented with multivitamin during pregnancy. By comparing to the RNI for pregnant women [25], median percentage of RNI for calcium was below 50%. Generally, the women had low activity level based on the medium Mets scores [23]. History of smoking or alcohol consumption was not reported among the women.

**Table 1:** Characteristics of the pregnant women (n = 102).

Variables	Second trimester	Third trimester
Gestational age, weeks [mean (SD)]	18.41 (3.71)	34.56 (1.84)
Age, years [n(%)]		
19–29	62 (60.8)	58 (56.9)
30–40	40 (39.2)	44 (43.1)
Parity [n(%)]		
0	25 (24.5)	
1	34 (33.3)	
≥2	43 (42.2)	
Employment [n(%)]		
Unemployed	22 (21.6)	
Working	80 (78.4)	
Pre-pregnancy BMI, kgm^−2^ [mean (SD)]	22.35 (4.27)	
Pre-pregnancy BMI categories [n(%)]		
<18.5	17 (16.7)	
18.5–24.99	59 (57.8)	
≥25.0	26 (25.5)	
Maternal BMI, kgm^−2^ [mean (SD)]	23.63 (4.19)	26.45 (4.03)
Maternal BMI categories [n(%)]		
<18.5	8 (7.8)	0
18.5–24.99	57 (55.9)	42 (41.2)
≥25.0	37 (36.3)	60 (58.8)
Multivitamin intake [n(%)]		
No	75 (73.5)	77 (75.5)
Yes	27 (26.5)	25 (24.5)
Dietary calcium, mg/day [median (IQR)]	486.81 (318.81–708.41)	485.31 (315.59–788.54)
Activity score, Mets [median (IQR)]	405.00 (120.00–1188.00)	480.75 (198.00–1253.63)
Activity status [n(%)]		
Low	64 (62.7)	59 (57.8)
Moderate	25 (24.5)	39 (38.2)
High	13 (12.7)	4 (3.9)

SD = standard deviation, IQR = interquartile range, BMI = body mass index, Mets = metabolic equivalents.

Maternal serum 25(OH)D levels during pregnancy are indicated in Table 2. In the second trimester, vitamin D deficiency [25(OH)D <50 nmol/L] was detected in 59.8% women. Of these, 3.9% women had severe vitamin D deficiency [25(OH)D <25 nmol/L] and 55.9% women had mild vitamin D deficiency [25(OH)D 25–<50 nmol/L]. The proportion of women having vitamin D deficiency decreased to 37.3% in the third trimester. More than one third of women had vitamin D insufficiency [25(OH)D 50–<75 nmol/L in the second trimester (34.3%) and third trimester (40.2%). Vitamin D sufficiency was found in 5.9% women in the second trimester and 22.5% women in the third trimester. Overall, mean levels of maternal serum 25(OH)D increased significantly from 48.45 nmol/L (SD 15.27) in the second trimester to 58.99 nmol/L (SD 20.43) in the third trimester of pregnancy (p<0.001).

**Table 2:** Maternal vitamin D levels during pregnancy (n = 102).

Serum 25(OH)D levels	Second trimester	Third trimester	p*
mean (SD)	48.45 (15.27)	58.99 (20.43)	<0.001
Vitamin D status, n (%)			
Severe deficiency (< 25 nmol/L)	4 (3.9)	0	
Mild deficiency (25–<50 nmol/L)	57 (55.9)	38 (37.3)	
Insufficiency (50–<75 nmol/L)	35 (34.3)	41 (40.2)	
Sufficiency (≥75 nmol/L)	6 (5.9)	23 (22.5)	
Total	102 (100.0)	102 (100.0)	

SD = standard deviation.

^*^p value obtained from paired t-test.

Table 3 presents the comparisons of maternal serum 25(OH)D levels during pregnancy based on several maternal factors. Levels of maternal 25(OH)D were not different according to pre-pregnancy BMI, maternal BMI, calcium intake and physical activity (p>0.05). On the other hand, women with multivitamin intake had significantly higher serum 25(OH)D than those without multivitamin intake in the second (p = 0.004) and third trimesters of pregnancy (p = 0.002). After adjusting for confounders, multivitamin intakes remained significantly associated with serum 25(OH)D levels during pregnancy (Table 4).

**Table 3:** Comparisons of maternal serum 25(OH)D levels according to maternal factors during pregnancy (n = 102).

		Serum 25(OH)D, nmol/L			
		Second trimester		Third trimester	
		Mean (SD)	p*	Mean (SD)	p*
Pre-pregnancy BMI, kgm^−2^	<18.5	52.41 (17.34)	0.166	61.59 (18.16)	0.807
	18.5–24.99	46.02 (15.11)		58.95 (21.53)	
	≥25	51.38 (13.67)		57.38 (19.79)	
Maternal BMI, kgm^−2^	<18.5	44.00 (17.05)	0.436	-	0.187
	18.5–24.99	47.60 (15.41)		62.19 (19.60)	
	≥25	50.73 (14.75)		56.75 (20.68)	
Multivitamin intake	No	45.31 (12.75)	**0.004**	55.51 (18.10)	**0.002**
	Yes	57.19 (18.32)		69.72 (23.66)	
Dietary calcium, mg/day	T1	45.21 (11.98)	0.318	60.59 (21.55)	0.719
	T2	49.91 (14.68)		59.68 (23.29)	
	T3	50.24 (18.40)		56.71 (16.18)	
Physical activity, Mets	Low	47.66 (15.97)	0.498	58.42 (22.69)	0.745
	Moderate/High	49.79 (14.11)		59.77 (17.06)	

BMI = body mass index, SD = standard deviation, T = tertile, Mets = metabolic equivalents.

^*^p values obtained from F tests using ANOVA. Bold print shows significant p<0.05.

Dietary calcium in the second trimester: T1 = <378 mg/day; T2 = 378–592 mg/day; T3 = >592 mg/day; Dietary calcium in the third trimester: T1 = <348 mg/day; T2 = 348–657 mg/day; T3 = >657 mg/day.

**Table 4:** Associations between maternal factors during pregnancy and serum 25(OH)D levels after adjusting for confounders^a^ (n = 102).

Maternal factors	Serum 25(OH)D in the second trimester, nmol/L^a^		Serum 25(OH)D in the third trimester, nmol/L^a^	
	β (95% CI)	p	β (95% CI)	p
Prepregnancy BMI, kgm^−2^	−0.06 (−0.74,0.62)	0.855	−0.40 (−1.37,0.57)	0.413
Maternal BMI, kgm^−2^	−0.20 (−0.90,0.49)	0.562	−0.68 (−1.69,0.33)	0.186
Multivitamin intake	9.16 (2.79,15.53)	**0.005**	13.65 (4.73,22.57)	**0.003**
Dietary calcium, mg/day	0.002 (−0.01,0.01)	0.706	−0.01 (−0.02,0.01)	0.281
Activity score, Mets (log unit)	−1.95 (−4.60,0.71)	0.148	2.18 (−2.20,6.55)	0.326

BMI = body mass index, Mets = metabolic equivalents. Bold print shows significant p<0.05.

a Adjusted for maternal age and gestational age.

Table 5 shows that mean levels of 25(OH)D in breast milk were 1.26 nmol/L, 1.18 nmol/L, 1.01 nmol/L and 1.16 nmol/L at delivery, 2, 6 and 12 months postpartum, respectively. As indicated in Table 6, there were no significant differences in two comparisons of each lactation stage (p>0.05).

**Table 5:** The 25(OH)D levels of breast milk.

Postnatal age	n	25(OH)D levels of breast milk, mean (95% CI)	
		nmol/L	IU/L
At delivery^a^	101	1.26 (1.09, 1.42)	20.16 (17.51, 22.81)
2 months	90	1.18 (1.09, 1.27)	19.21 (17.70, 20.73)
6 months	69	1.01 (0.99, 1.04)	16.39 (15.93, 16.86)
12 months	49	1.16 (1.02, 1.31)	18.80 (16.41, 21.19)

a Between 1 and 14 days postpartum.

**Table 6:** Comparison of breast milk 25(OH)D levels at different stages during the first year of lactation.

Comparison	25(OH)D levels of breast milk (nmol/L), mean difference (95% CI)	p
At delivery – 2 months	0.17 (−0.29, 0.63)	>0.95
At delivery – 6 months	0.31 (−0.09, 0.72)	0.228
At delivery – 12 months	0.19 (−0.27, 0.64)	>0.95
2 months – 6 months	0.15 (−0.06, 0.35)	0.307
2 months – 12 months	0.02 (−0.27, 0.31)	>0.95
6 months – 12 months	−0.13 (−0.34, 0.09)	0.657

Repeated measures ANOVA within group analysis was applied followed by pairwise comparison with confidence interval adjustment.

The associations between maternal serum and breast milk 25(OH)D levels are shown in Table 7. There was a positive association between serum 25(OH)D level in the second trimester and breast milk 25(OH)D level at delivery (β = 0.002, p = 0.026); whereas breast milk 25(OH)D levels at 2, 6 and 12 months postpartum were not associated with maternal values in the second trimester. Similarly, no association was observed between levels of 25(OH)D in maternal serum in the third trimester and breast milk.

**Table 7:** Associations between maternal serum and breast milk 25(OH)D levels after adjusting for confounders.^a^

Maternal serum 25(OH)D levels, nmol/L	25(OH)D levels of human milk, nmol/L (log)							
	At delivery		2 months		6 months		12 months	
	β	p	β	p	β	p	β	p
Second trimester	0.002 (0,0.003)	**0.026**	0 (−0.001,0.001)	0.591	0 (−0.001,0.001)	0.639	−0.001 (−0.002,0.001)	0.389
Third trimester	0.001 (0,0.002)	0.098	0 (−0.001,0.001)	0.622	0 (−0.001,0.001	0.241	0.001 (−0.001,0.002)	0.424

Bold print shows significant p<0.05.

a Adjusted for maternal age and prenatal multivitamin intake.

## Discussion

This study provides the first data on vitamin D status in Malaysian pregnant women. Less than 4% of women were shown to have severe vitamin D deficiency (<25 nmol/L) during pregnancy, suggesting that this population may have a low risk of developing maternal osteomalacia [19]. However, 40% to 60% of women were found to have vitamin D deficiency (<50 nmol/L), which may impose negative effects on immune health, bone health, neural development and increase the risk of fracture in the offspring during childhood [26]. Compared to other studies in the Asian region, a relatively lower proportion of maternal vitamin D deficiency was observed in our study. Recent studies in India [27] and China [28] revealed that 74% and 97% of pregnant women had serum 25(OH)D below 50 nmol/L. Although optimal concentrations of maternal 25(OH)D at different gestational periods are not known, there is emerging evidence showing that low vitamin D status (<50 nmol/L) is currently common in pregnant women [8], [29].

Despite living in a country with an abundance of sunshine, the majority of women in Malaysia had inadequate levels of vitamin D (<75 nmol/L). This phenomenon could be attributable to the combined influences of darker skin, cultural avoidance of sun exposure and clothing style that cover most of the body while outdoors [29], [30]. Green et al. [17] and Moy and Bulgiba [30] found that the means of serum 25(OH)D in Malay women of childbearing age were 43 nmol/L and 36 nmol/L, respectively. Rahman et al. [31] indicated that Malay postmenopausal women had serum 25(OH)D at the mean level of 44 nmol/L. A recent study which was conducted among urban and rural women in Malaysia showed that 25(OH)D concentration was at the mean of 65 nmol/L [32]. Our results which showed maternal serum 25(OH)D levels at 48 nmol/L and 59 nmol/L were comparable to these reported values.

In general, the circulating 25(OH)D levels during gestation do not change [33], although one report from Spain noted a significant decline in 25(OH)D levels by the third trimester of pregnancy [13]. However, the opposite effect was observed in our study. The maternal 25(OH)D levels significantly increased from the second to the third trimesters of pregnancy. This result is in agreement with a Thai study which showed significant increment of maternal serum 25(OH)D levels during the course of pregnancy [14]. Even after removing women who were using vitamin supplementation throughout gestation, the current findings remained similar (data not shown). It is suggested that such enhanced levels of serum 25(OH)D are unlikely to be caused by altered vitamin D metabolism during pregnancy. As reported previously, 25(OH)D levels are relatively unaffected by pregnancy despite there is an increase of calcitriol level during pregnancy and passage of 25(OH)D across the placenta to the fetus [10]. Instead, it could be due to the changes in lifestyle factors towards the end of pregnancy, such as higher vitamin-fortified milk consumption [14] or more sunlight exposure [10] which represents the key factor on influencing vitamin D status. Otherwise, genetic factors and changes in adipose tissue deposition during pregnancy may also effect maternal serum 25(OH)D levels [10], [14]. Further investigations are required in this area.

Instead of vitamin D supplementation alone, pregnant women in Malaysia are commonly supplemented with multivitamins which contain 400 IU of vitamin D. These multivitamin intakes were consistently associated with higher maternal serum 25(OH)D levels in the second and third trimesters of pregnancy, which is in line with previous reviews that indicated the positive effect of vitamin D supplementation on increasing serum vitamin D status [7], [34]. On the other hand, maternal BMI, pre-pregnancy BMI, dietary calcium intake and physical activity were not significantly associated with serum 25(OH)D during pregnancy. It may be that most women in this study had normal BMI status, did not meet the RNI for calcium intake and were not physically active enough to exert an effect on the levels of maternal serum 25(OH)D during pregnancy. Supportive evidence has been reported in a few studies. Nurbazlin et al. [32] showed that body fat percentage did not appear as a significant contributor to 25(OH)D level. Rahman et al. [31] reported a non-significant association between physical activity and 25(OH)D level. Green et al. [17] found that the relationship between 25(OH)D and parathyroid hormone (PTH) concentrations did not differ between women with low, medium or high calcium intake.

As reviewed by Dawodu and Tsang [19], most studies of breast milk vitamin D levels were reported more than two decades ago from North America and Europe. Assessment of vitamin D in breast milk among Asians has not previously been reported. In general, the mean of breast milk total vitamin D activity in healthy lactating women ranged from 10 to 80 IU/L [19]. The 25(OH)D has been shown to contribute to about 33 IU/L of vitamin D activity in breast milk [35], which is relatively higher than our findings that ranged from 16 to 20 IU/L. These levels of breast milk 25(OH)D did not appear to change significantly during the first year of lactation. Since vitamin D levels in breast milk are dependent on maternal vitamin D status [36], the current result is therefore reflecting that maternal serum vitamin D levels remained similar during lactation. A recent study in Malawi indicated that vitamin D was undetectable in breast milk at delivery and 2 months postpartum [37]. Earlier reports have shown that milk of healthy lactating women contains relatively small amount of vitamin D and is a poor source of vitamin D for the infants [10], [19].

The 25(OH)D levels in the milk from mothers in our study are closer to that observed in dark skinned than white skinned women [38], which may be attributable to increased skin pigmentation that reduces the skin's ability to produce vitamin D from sunlight. Otherwise, it may reflect low intake of vitamin D or sunlight exposure among the women. Such low levels of vitamin D in breast milk might result in low intake of vitamin D among breastfed infants as compared to the recommended intake of 400 IU/d of vitamin D [10]. It is therefore expected that a significant proportion of exclusively breastfed infants are at risk of vitamin D deficiency. According to Balasubramanian and Ganesh [18], there is rising recognition of vitamin D deficiency during infancy and this has become a public health and pediatric problem in many countries.

It has been reported that cord blood 25(OH)D level was correlated positively with maternal value [27], [39], indicating infant vitamin D stores at birth rely on maternal vitamin D status during pregnancy. An early study by Hollis et al. [40] showed that all vitamin D compounds in breast milk were associated with maternal levels during lactation. Our finding provides additional evidence that breast milk 25(OH)D level at delivery was associated with maternal serum 25(OH)D in the second trimester of pregnancy instead of third trimester of pregnancy. This situation may be explained by the mechanism of breast milk vitamin D production. It is possible that there could be some lag between elevated levels of vitamin D in the blood and breast milk whereby times are required to enable vitamin D from the adipose tissue to be made available for milk production. This suggests that maternal vitamin D status during mid gestation is potentially affecting vitamin D level in breast milk at delivery and thereby, influencing vitamin D status of the breastfed infants in their early life. This situation should be of concern, especially if the mother plans to exclusively breastfeed. Currently, there are ongoing research activities which focus on high-dose maternal vitamin D supplementation alone during lactation to increase vitamin D level in breast milk [19].

The present results may not be generalizable to all pregnant women in Malaysia as convenience sampling technique was adopted. Only Malay women within a particular region with specific local ethnic practice were recruited. Also, other factors associated with vitamin D status were not measured, such as PTH and sunlight exposure, as well as influencing factors during lactation which might affect human milk vitamin D levels. Therefore, the findings that indicated a positive association between maternal serum and breast milk vitamin D levels shall be cautiously interpreted as there was limited adjustment for confounders. Nevertheless, this study represents the first study in Malaysia to investigate vitamin D status in pregnant women and breast milk. The present study adds to the small number of studies that have reported vitamin D changes during pregnancy and lactation. These data are important for future intervention studies which aim to increase vitamin D status in pregnant women, lactating women or breastfed infants. Furthermore, application of a longitudinal approach reduces inter-individual variation in studying vitamin D changes during pregnancy and lactation. Also, vitamin D levels in this study were measured using gold standard HPLC-MS method which has greater specificity and increased results precision compared with immunoassays and HPLC-UV methods [41].

In conclusion, vitamin D insufficiency was common in Malay pregnant women. Maternal serum 25(OH)D in the second trimester of pregnancy was potentially associated with 25(OH)D level in breast milk at delivery, which is expected to affect the vitamin D status of the breastfed infant in early life. This result suggests that intervention to optimize vitamin D in breast milk should be started early in pregnancy, rather than during the lactation period.
